# Bladder Herniation through the Obturator Foramen Treated with Laparoscopic Transabdominal Preperitoneal Repair Using a Contralateral 3D Max Mesh: A Case Report

**DOI:** 10.70352/scrj.cr.25-0640

**Published:** 2025-12-18

**Authors:** Ryozan Naito, Keisuke Ieta, Momoka Arai, Shigeo Maki, Katsuya Osone, Masaki Suzuki, Yohei Miyamae, Keitaro Hirai, Ichiro Sakamoto, Hiroshi Saeki

**Affiliations:** 1 Department of Surgery, Takasaki General Medical Center, Takasaki, Gunma, Japan; 2 Department of General Surgical Science, Gunma University Graduate School of Medicine, Maebashi, Gunma, Japan

**Keywords:** bladder hernia, obturator hernia, transabdominal preperitoneal (TAPP)

## Abstract

**INTRODUCTION:**

Obturator hernia is an uncommon condition. With the widespread adoption of laparoscopic surgery, incidental detection of obturator hernias has become increasingly frequent. Bladder herniation through the obturator foramen is extremely rare, with only 14 cases reported to date. Herein, we describe a rare case of obturator bladder hernia successfully repaired via the laparoscopic transabdominal preperitoneal (TAPP) approach, highlighting a novel technical modification.

**CASE PRESENTATION:**

A 74-year-old female was diagnosed with an obturator bladder hernia. The patient initially presented to the hospital with hematochezia and was diagnosed with ischemic colitis. Initial CT revealed an obturator hernia in the small intestine. The following day, CT revealed an obturator bladder hernia, although she had no symptoms related to an obturator bladder hernia. Subsequent CT at our hospital established a diagnosis of obturator bladder hernia. The patient underwent laparoscopic obturator hernia repair via the TAPP approach. A contralateral 3D MAX MID Anatomical Mesh (Bard; Warwick, RI, US) was deployed. The mesh was rotated 180° around its medial axis and positioned with the convex surface facing the abdominal wall to achieve an optimal anatomical fit. The operative time was 148 minutes, with an estimated blood loss of 10 mL. The postoperative course was uneventful, and the patient was discharged on the 3rd POD.

**CONCLUSIONS:**

Currently, there is no clear consensus regarding the surgical indications for obturator bladder hernia. In the present case, although the patient was asymptomatic, daily changes in hernia contents raised concerns regarding potential small bowel incarceration, prompting surgical intervention. Our novel modification of the TAPP approach involved the placement of a contralateral 3D MAX mesh rotated 180° around its medial axis. This adjustment preserved the anatomical curvature of the mesh, facilitated secure fixation, and ensured adequate coverage of the obturator foramen and femoral ring.

## Abbreviations

MPO
: myopectineal orifice
TAPP
: transabdominal preperitoneal

## INTRODUCTION

Obturator hernia is an uncommon condition, with an incidence of approximately 0.073% of all surgically repaired hernias at the Mayo Clinic.^1)^ With the widespread adoption of laparoscopic surgery, incidental detection of obturator hernias has become increasingly frequent. Recent studies have reported an incidence of 6.82%, which is substantially higher than that previously reported.^2)^ Bladder herniation through the obturator foramen (obturator bladder hernia) is extremely rare, with only 14 cases reported to date. Among these, only 3 cases were treated using the laparoscopic TAPP approach.^3–5)^ Herein, we describe a rare case of obturator bladder hernia successfully repaired via the TAPP approach, highlighting a novel technical modification.

## CASE PRESENTATION

**Fig. 1:**
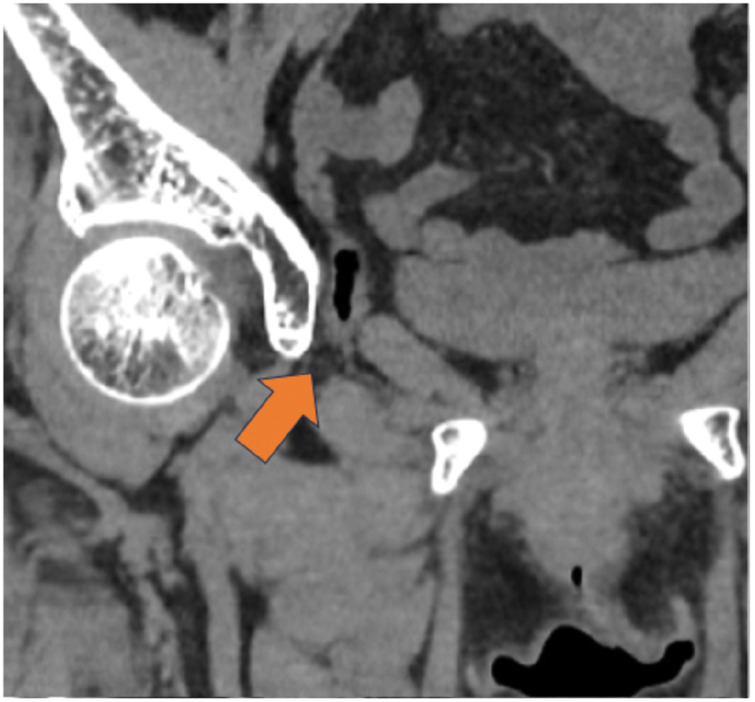
The initial CT scan performed at the referring hospital. Arrow: herniation of the small intestine through the obturator foramen.

**Fig. 2:**
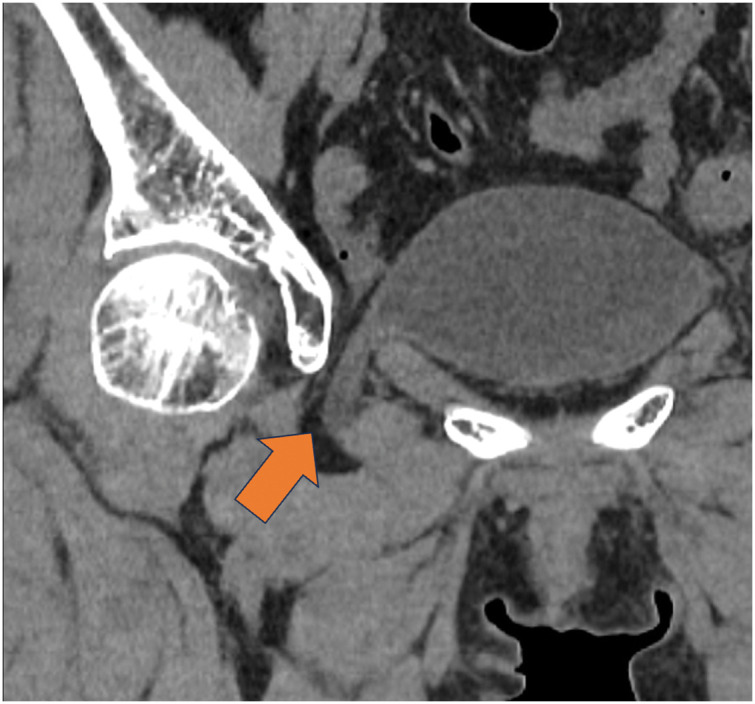
The CT scan performed on the following day at the referring hospital. Arrow: bladder herniation through the obturator foramen.

**Fig. 3:**
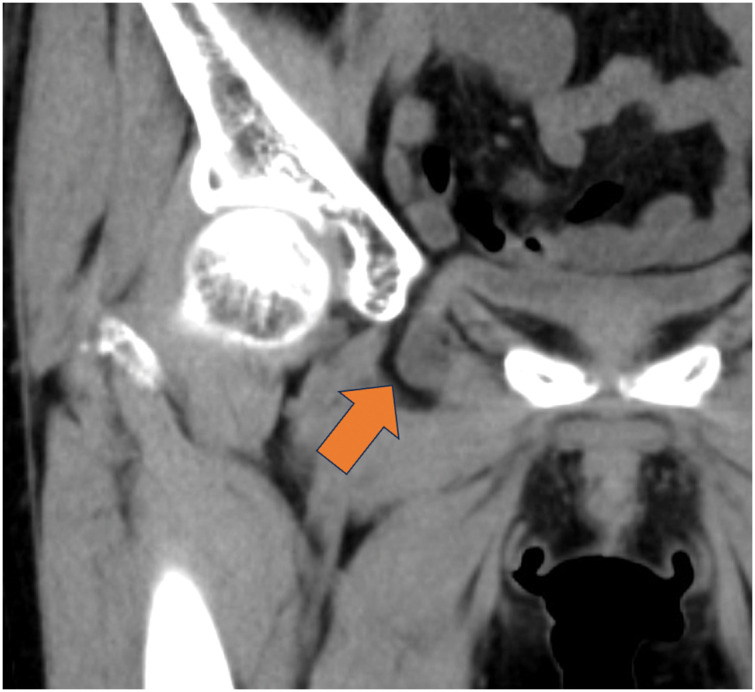
The CT scan performed at our hospital. Arrow: bladder herniation through the obturator foramen.

A 74-year-old female was diagnosed with an obturator bladder hernia and was referred to our department for surgical repair. The patient initially presented to the hospital with hematochezia and was diagnosed with ischemic colitis. CT performed at the time of diagnosis incidentally revealed an obturator hernia in the small intestine (**Fig. 1**). On the following day, a repeat CT demonstrated bladder herniation through the obturator foramen (**Fig. 2**). Thus, she was subsequently referred to our hospital for further evaluation and management. Her ischemic colitis resolved conservatively. The patient’s height and weight were 151.7 cm and 45.3 kg, respectively, with a BMI of 19.7. She had no symptoms related to an obturator bladder hernia. Her medical history included uterine leiomyoma and appendicitis, for which she had previously undergone laparoscopic myomectomy and appendectomy, respectively. She was a nonsmoker and reported occasional alcohol consumption. CT at our hospital confirmed bladder herniation through the obturator foramen, establishing a diagnosis of obturator bladder hernia (**Fig. 3**). After providing a detailed explanation of her condition and associated risks, the patient consented to surgical treatment.

**Fig. 4:**
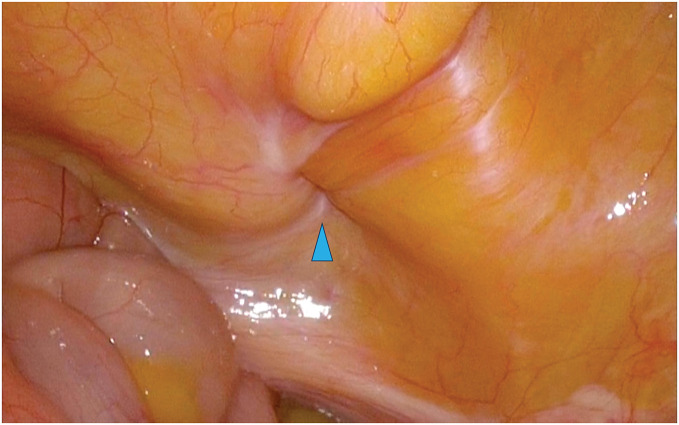
The right obturator hernia observed from within the abdominal cavity. Arrowhead: the obturator foramen.

**Fig. 5:**
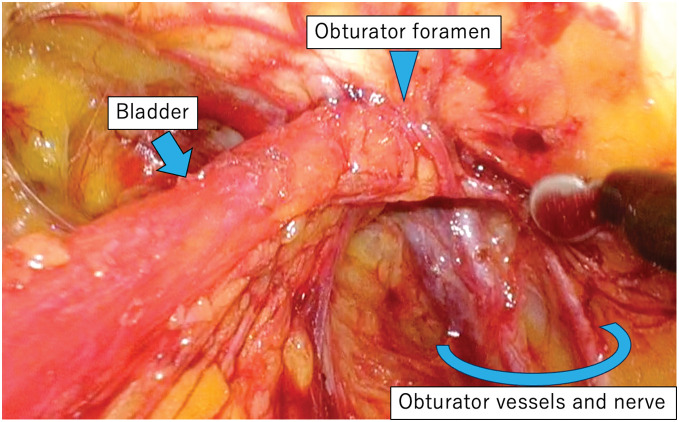
Bladder herniation through the right obturator foramen.

**Fig. 6:**
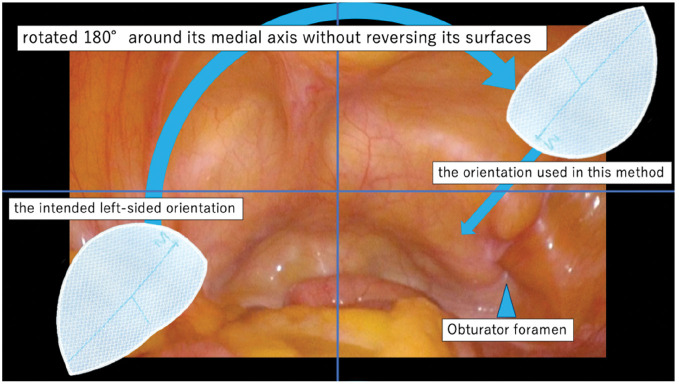
Schematic illustration of the present technique.

**Fig. 7:**
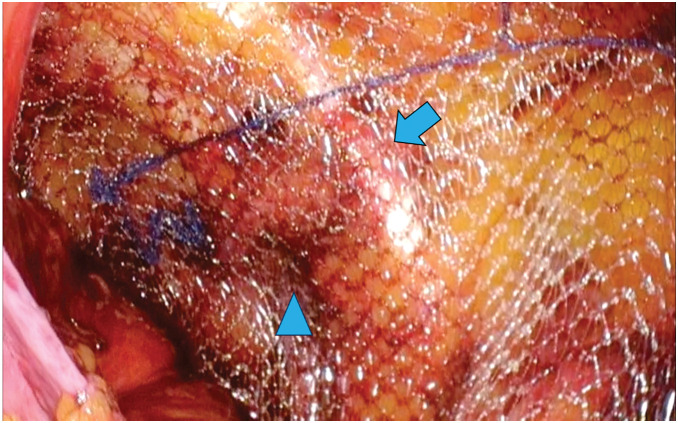
The operative field after mesh deployment. Arrowhead: the obturator foramen. Arrow: the cooper’s ligament.

The patient underwent laparoscopic obturator hernia repair via the TAPP approach. A 12-mm camera port at the umbilicus and two 5-mm ports on the bilateral flanks were installed. Dense adhesions due to a previous appendectomy were observed in the right lower quadrant, obscuring the intended site for the right-hand port. Prolonged and careful dissection was required to separate the adhesions. A right obturator hernia with a 1 × 1-cm defect was observed, which also contained a space large enough to accommodate the surgical instruments (**Fig. 4**). A left side obturator hernia with 1.5 × 1.5-cm defect was also observed, but other hernia foramen were not observed. The peritoneum was incised from the lateral aspect of the right internal inguinal ring to the medial aspect of the inferior epigastric vessel. Subsequently, dissection was performed from the medial side of the inferior epigastric vessels into the prevesical (Retzius) space, and the same anatomical plane was further developed (**Fig. 5**). The obturator nerve and vessels were preserved and the bladder was successfully reduced from the obturator foramen into the abdominal cavity. Following extensive peritoneal dissection, a left-sided 3D MAX MID Anatomical Mesh (size, L; Bard; Warwick, RI, US) was deployed. The left-sided mesh was rotated 180° around its medial axis and positioned with the convex surface facing the abdominal wall to achieve an optimal anatomical fit (**Figs. 6** and **7**). The left side was repaired in a similar fashion with a right-sided mesh, and the peritoneum on both sides was closed with sutures, thereby completing the operation. The operative time was 148 minutes, with an estimated blood loss of 10 mL. The postoperative course was uneventful, and the patient was discharged on the 3rd POD. After a short-term follow-up, no complications were noted.

## DISCUSSION

Herein, we report a case of an obturator bladder hernia that was successfully treated using a TAPP approach with a slight modification. Articles concerning obturator bladder hernia were identified through a database search of PubMed and the Japan Medical Abstracts Society (Ichushi), using the keywords “obturator hernia” and “bladder,” and included publications up to August 2025. Bladder herniation through an obturator hernia is an extremely rare condition, with only 14 cases reported to date (**Table 1**).^3–16)^ Among these, 13 patients were female, with a mean age of 78 years (range 60–96 years). Eight patients presented with symptoms, such as thigh pain, pollakiuria, or residual urine. Nine patients underwent surgical repair, including 4 open laparotomies and 5 laparoscopic procedures.

**Table 1:** Summary of previous reports

No	Year	Author	Sex	Age	Surgical procedure	Side	Other concomitant hernias	Related symptoms with bladder herniation
1	1901	Gladstone^6)^	F	78	–	Left	Right obturator	Thigh pain
2	1976	McCarthy^7)^	M	60	Open	Right	–	Thigh pain
3	1997	Fritz^8)^	F	86	Open	Right	–	Abdominal pain, urinary retention
4	2008	Lopez^9)^	F	66	Laparoscopic	Right	–	Pollakiuria, urinary tract infection
5	2009	Kaneta^10)^	F	73	–	Left	–	–
6	2009	Kikkawa^11)^	F	96	–	Right	–	–
7	2012	Ogata^12)^	F	80s	–	Bilateral	–	–
8	2016	Watanabe^13)^	F	77	Open	Left	–	Thigh pain
9	2018	Inoue^14)^	F	78	Open	Right	–	Lower abdominal pain
10	2018	Mei^3)^	F	88	TAPP	Right	Right inguinal, right femoral	Pollakiuria, residual urine
11	2019	Yuba^15)^	F	79	TEP	Left	Left inguinal, right obturator	–
12	2023	Kuhara^4)^	F	66	TAPP	Left	Left inguinal	Pollakiuria, leg pain
13	2025	Scott^16)^	F	80	–	Right	–	–
14	2025	Naito^5)^	F	70	TAPP	Right	Right femoral	–

F, female; M, male; TAPP, transabdominal preperitoneal; TEP, totally extraperitoneal

Currently, there is no clear consensus regarding the surgical indications for obturator bladder hernia. Surgical intervention is generally considered reasonable when patients develop pain or urinary disturbances such as frequent urinary tract infections.^9)^ In the present case, the patient was asymptomatic; however, the hernia contents varied on a daily basis, raising concerns about possible small bowel incarceration. To our knowledge, no previous report has described daily changes in hernia content as observed in the present case. Therefore, after a thorough discussion with the patient, surgical intervention was performed.

In the 3 previously reported cases of obturator bladder hernia repaired using a TAPP approach,^3–5)^ no specific technique for managing bladder herniation was described. Cadaveric studies indicated that focal adhesions at the obturator foramen and dilation of the obturator canal are chronic markers.^16)^ By contrast, in our case, the bladder herniation did not adhere to the obturator foramen; therefore, no additional maneuvers were required. This observation may explain the daily variations in hernia contents noted in the present case. Intraoperative observation of the abdominal cavity revealed that the present case represented a paraperitoneal type of bladder hernia.^17)^ Notably, the same foramen also contained a space large enough to accommodate the surgical instruments, indicating that small bowel incarceration could not be completely excluded. This finding suggests that small-bowel herniation can occur concurrently with bladder herniation through the obturator foramen.

The feasibility and effectiveness of laparoscopic surgery for the management of obturator hernias have been well-documented.^18)^ The flat mesh is typically used in TAPP repair for obturator hernias, but Chiba et al. have described a TAPP approach for obturator hernia using a 3D MAX mesh.^19)^ The 3D MAX mesh is a shape-memory prosthesis originally designed for inguinal hernia repair, with an anatomically curved design intended to fit the groin area. In this report, the mesh was positioned with reversed anterior and posterior surfaces. This technique is based on the finding that, in thin elderly women, the angle between the obturator fascial plane and the Hesselbach triangle at Cooper’s ligament is approximately 160°. However, with this placement, the mesh protrudes into the intra-abdominal cavity, thereby diminishing the intended advantage of the anatomical curvature. Additionally, the slack of the mesh is a disadvantage during the tracking procedure because it can lead to slippage or distortion.

Uniquely, in the present case, we placed a mesh originally designed for the contralateral side after rotating it 180° around its medial axis without reversing its surface. This novel adjustment preserved the anatomical curvature of the mesh and facilitated a straightforward and secure fixation procedure. With this technique, adequate coverage of the obturator foramen and femoral ring can be achieved; however, coverage of the MPO remains insufficient. Although precise data on complication rates are lacking, obturator hernia and femoral hernia share common risk factors, including advanced age, female sex, low BMI, and multiparity.^20–22)^ By contrast, inguinal hernia is more frequently observed in males.^23)^ Therefore, routine coverage of the MPO is not necessary. Rather, the need for additional mesh placement should be assessed individually for each case. In the present case, because the risk of an inguinal hernia was deemed low, MPO coverage was not performed.

## CONCLUSIONS

In conclusion, we report a rare case of obturator bladder hernia that was successfully treated using the laparoscopic TAPP approach. Obturator bladder hernia is exceptionally uncommon, and there is no clear consensus on its surgical management. In the present case, although the patient was asymptomatic, daily changes in hernia contents raised concerns regarding potential small bowel incarceration, prompting surgical intervention. Our novel modification involved the placement of a contralateral 3D MAX mesh rotated 180° around its medial axis. This adjustment preserved the anatomical curvature of the mesh, facilitated secure fixation, and ensured adequate coverage of the obturator foramen and femoral ring.

### Patient perspective

The patient was reassured by the clear explanation of the risks and was satisfied with the decision for surgical repair. She expressed relief with the smooth postoperative course and her ability to resume daily activities without difficulty.

## SUPPLEMENTARY MATERIAL

Mesh placement procedure in the present case.

## DECLARATIONS

### Availability of data and materials

Data sharing is not applicable to this article as no new data were created or analyzed in this study.
